# Full-Duplex Relay for Millimeter Wave Vehicular Platoon Communications

**DOI:** 10.3390/s20216072

**Published:** 2020-10-26

**Authors:** Jong-Ho Lee, Jiho Song

**Affiliations:** 1 School of Electronic Engineering, Soongsil University, Seoul 06978, Korea; jongho.lee@ssu.ac.kr; 2 School of Electrical Engineering, University of Ulsan, Ulsan 44610, Korea

**Keywords:** vehicle communication, platoon, millimeter wave, full-duplex, power allocation

## Abstract

The safety and consistency of platoon-based driving are guaranteed via reliable communication between vehicles in a platoon. In this paper, we propose to exploit a full-duplex (FD) relay vehicle for millimeter-wave vehicular communications in platoon-based driving. Considering that a lead vehicle broadcasts information to all vehicles in a platoon, we consider two power allocation problems—maximizing broadcast information rates with a power constraint and minimizing power consumption with a quality-of-service constraint. In particular, for a four-vehicle platoon communication, we derive closed-form solutions for optimal power allocation and present numerical results to verify the performance of the proposed FD relay vehicles.

## 1. Introduction

Autonomous vehicular platoon systems are a promising service for intelligent transport systems owing to their strong potential to significantly improve road capacity and energy efficiency [1,2,3,4]. In platoon-based driving, a group of vehicles operate cooperatively and coordinate their speed and distance. Each vehicle in a platoon follows its preceding vehicle, maintaining an almost constant and small distance. As the distance between the vehicles in a platoon can be maintained to be small, the road capacity can be enhanced and traffic congestion can be reduced. Furthermore, energy consumption is expected to be reduced because the air drag can be minimized in a series of succeeding vehicles.

Cooperative adaptive cruise control (CACC) and vehicle-to-vehicle (V2V) communication are the enabling technologies for autonomous vehicular platoon systems [3]. The objective of CACC is to control the distances between vehicles using information collected by sensors and V2V communication links. Through V2V communication, vehicles in a platoon share both safety-related information, such as speed, position, and acceleration, and non-safety-related information, such as weather conditions and media access for the stabilization of platoon [5,6]. As the performance of V2V communication is critical for the stability of autonomous vehicular platoon systems, there have been extensive research efforts on the interference management, security, and latency of V2V communication links [7,8,9].

As massive automotive sensors and radars in a vehicle collect lots of data, the amount of information shared between vehicles increases extensively [10]. As in the fifth generation (5G) of wireless systems, millimeter wave (mmWave) communication has been a promising technique in vehicular communications to meet the communication demand for sharing lots of sensing data between vehicles [11,12,13,14,15]. The large bandwidth of mmWave signals can be exploited to facilitate high speed communication between vehicles. Further, the small wavelength of mmWave signals allows vehicles to place a large number of antennas in a small area to achieve high antenna array gains.

In this study, we consider that a lead vehicle, which is the first vehicle of a platoon, broadcasts the information to all vehicles in that platoon using mmWave signals. The vehicle immediately behind the lead vehicle can have a line-of-sight (LOS) link from the lead vehicle, whereas the LOS link is not guaranteed for other vehicles due to the blockage caused by their preceding vehicles. Since the blockage of the LOS link causes severe propagation loss in mmWave frequencies [16,17], we assume that some vehicles in a platoon operate as a relay vehicle for reliable platoon communications. It is noteworthy that exploiting an unmanned aerial vehicle (UAV) is also an attractive solution because the LOS link between the UAV and each vehicle in the platoon can be guaranteed when the UAV is well located [18].

Furthermore, let us assume that a transmit antenna is placed on the rear of the vehicle, whereas a receive antenna is located in front of the vehicle. Subsequently, we can obtain good isolation between the transmitting and receiving antennas in a vehicle [19,20]. Moreover, the directional properties and poor scattering characteristics of mmWave frequencies make it impossible for a vehicle to receive its own transmitting radio signals. Based on these observations, we suggest that relay vehicles in a platoon perform full-duplex (FD) operation to receive the radio signals transmitted by the preceding vehicles and simultaneously forward them to the following vehicles at the same frequency.

Conventional half-duplex (HD) relaying techniques have been extensively studied in V2V communications [21,22,23], whereas research on the use of FD relaying techniques is limited [24,25,26]. In particular [25], analyzed the outage probabilities and throughput of the V2V communication link assisted by the FD relay vehicle in dense multi-lane highway environments. In [26], FD relay vehicles were utilized in vehicular platoon communications, and FD relay vehicles were shown to provide enhanced packet delivery ratios and reduced physical layer latency compared with conventional HD relay vehicles. However, there were no considerations for transmit power optimization and relay vehicle selection.

In this study, we investigate power allocation problems from the viewpoint of safety and consistency for exploiting FD relay vehicles in vehicular platoon communications. The safety of platoon-based driving is guaranteed when the safety-related information is broadcast without errors through reliable communication links. As the broadcast information rate increases, the communication links become more reliable. Herein, we consider a power allocation problem to maximize broadcast information rates with a power constraint. On the contrary, sustainable vehicular communication guarantees the consistency of platoon-based driving. To improve the sustainability of vehicular platoon communications, the power consumption for vehicular communications should be minimized. Therefore, we investigate a power allocation problem to minimize power consumption with a quality-of-service (QoS) constraint.

We present that the optimal power allocation to maximize broadcast rates with a power constraint can be obtained via linear programming. Further, the power allocation problem to minimize power consumption with a QoS constraint is shown to be a quadratically constrained linear programming problem. Subsequently, we focus on platoon communication with four vehicles and derive closed-form solutions for optimal power allocation. Using the closed-form solutions, we also suggest a relay-vehicle selection approach. Numerical results are presented to demonstrate that the FD operation in vehicular platoon communications provides a significant performance improvement over the conventional HD operation. The key notations used in this study are summarized in Table 1.

## 2. System Model and Problem Formulation

**Figure 1:**
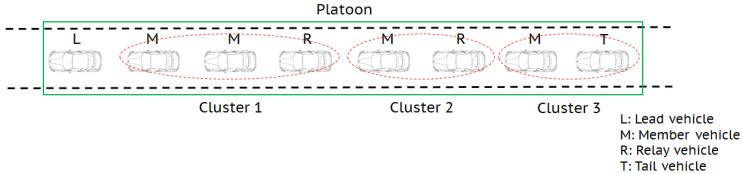
Vehicle platoon communication model (Λ=8, K=3, $N_{1}=2$, and $N_{2}=N_{3}=1$).

A vehicular platoon communication model is considered, as shown in Figure 1, where the lead vehicle sends broadcast information to all vehicles in a platoon [6]. Relay vehicles in a platoon assist the lead vehicle by receiving the information from the preceding vehicle and forwarding it to the following vehicles. The vehicle located at the end of the platoon is the tail vehicle. The other vehicles are referred to as member vehicles.

Let us consider a platoon with Λ vehicles. The first vehicle plays the lead vehicle, whereas the last vehicle is a tail vehicle. Each vehicle located in the middle of the platoon can be a relay vehicle or a member vehicle. Let us assume that K−1 vehicles are chosen as FD relay vehicles. The relay vehicles are indexed using *k* with k=1,2,⋯,K−1. The lead and tail vehicles are indexed by 0 and *K*, respectively. The received signal at the *k*th relay vehicle can be given as

(1) $$y_{k}=h_{k-1,k}\sqrt{P_{k-1}}s_{k-1}+\sum_{q=0}^{k-2}h_{q,k}\sqrt{P_{q}}s_{q}+z_{k},$$

where $h_{q,k}$ is the channel coefficient between the *q*th relay vehicle and the *k*th relay vehicle. The noise $z_{k}$ is assumed to be a complex additive white Gaussian with zero mean and variance $\sigma^{2}$, and $P_{k}$ denotes the transmit power of the *k*th relay vehicle. $P_{0}$ denotes the transmit power of the lead vehicle. The received signal at the tail vehicle can be given as Equation (1) with k=K.

In Equation (1), $s_{q}$ denotes the information symbol transmitted by the *q*th relay vehicle. Let $s_{0}$ be the information symbol transmitted by the lead vehicle. As the relay vehicle also has to hear the message from the lead vehicle, it decodes the signal and forwards it to those following it, indicating the decode-and-forward operation [27]. When the *q*th FD relay operates in the decode-and-forward mode, $s_{q}$ becomes the delayed version of $s_{q-1}$ induced by the decoding process and propagation delay. Therefore, the first term on the right-hand side of Equation (1) is the desired signal for the *k*th relay vehicle while the second term denotes the signals from the lead vehicle and the first k−2 relay vehicles, which can be considered inter-symbol interference. We, herein, assume that the self-interference induced by the FD operation is negligible, as mentioned in Section 1.

### 2.1. Vehicle Clustering

As shown in Figure 1, each relay vehicle and the tail vehicle form their own cluster. Each member vehicle belongs to a cluster formed by the nearest relay vehicle behind it. The member vehicle that has no relay vehicle behind it belongs to the cluster of the tail vehicle. Subsequently, we have *K* clusters in the platoon and the received signal at the *n*th member vehicle in the *k*th cluster can be expressed similar to Equation (1) given as

(2) $$y_{k:n}=h_{k-1,k:n}\sqrt{P_{k-1}}s_{k-1}+\sum_{q=0}^{k-2}h_{q,k:n}\sqrt{P_{q}}s_{q}+z_{k:n},$$

where $h_{q,k:n}$ is the channel coefficient between the *q*th relay vehicle and the *n*th member vehicle in the *k*th cluster, and $z_{k:n}$ is also assumed to follow a complex Gaussian distribution with zero mean and variance $\sigma^{2}$. Moreover, $N_{k}$ denotes the number of member vehicles in the *k*th cluster with $n=1,2,\cdots ,N_{k}$, and the total number of vehicles in the platoon can be given as $\Lambda =K+1+\sum_{k=1}^{K}N_{k}$.

### 2.2. Power Allocation Problems

From Equations (1) and (2), we can compute the information rates at the *k*th relay vehicle and the *n*th member vehicle in the *k*th cluster given as

(3) $$\begin{array}{ccc} R_{k} & = & \log_{2}\left(1+\frac{\alpha_{k-1,k}P_{k-1}}{1+\sum_{q=0}^{k-2}\alpha_{q,k}P_{q}}\right),\\ R_{k:n} & = & \log_{2}\left(1+\frac{\alpha_{k-1,k:n}P_{k-1}}{1+\sum_{q=0}^{k-2}\alpha_{q,k:n}P_{q}}\right), \end{array}$$

respectively, where we define $\alpha_{q,k}=\frac{|h_{q,k}{|}^{2}}{\sigma^{2}}$ and $\alpha_{q,k:n}=\frac{|h_{q,k:n}{|}^{2}}{\sigma^{2}}$. As all vehicles in a platoon should correctly decode the message from the lead vehicle, the broadcast information rate should be determined by

(4) $$R=\min\left\{R_{k},R_{k:n};k=1,2,\cdots ,K,\;n=1,2,\cdots ,N_{k}\right\}.$$

In this study, we consider two power allocation problems to determine $P_{k}$ with k=0,1,⋯,K−1. The first one maximizes the broadcast rate with a power constraint, indicating that the overall power consumption is limited to $\bar{P}$ as follows:

(5) $$\begin{array}{c} \begin{array}{cc} \max_{P_{0},P_{1},\cdots ,P_{K-1}} & \min\left\{\frac{\alpha_{k-1,k}P_{k-1}}{1+\sum_{q=0}^{k-2}\alpha_{q,k}P_{q}},\frac{\alpha_{k-1,k:n}P_{k-1}}{1+\sum_{q=0}^{k-2}\alpha_{q,k:n}P_{q}};\;k=1,\cdots ,K,\;n=1,\cdots ,N_{k}\right\}\\ s.t. & P_{0}+P_{1}+\cdots +P_{K-1}\leq \bar{P},\\ & P_{k}\geq 0,\;k=0,\cdots ,K-1. \end{array} \end{array}$$

On introducing an auxiliary variable τ, Equation (5) can be equivalently rewritten as

(6) $$\begin{array}{c} \begin{array}{cc} \max_{P_{0},P_{1},\cdots ,P_{K-1},\tau } & \tau \\ s.t. & \frac{\alpha_{k-1,k}P_{k-1}}{1+\sum_{q=0}^{k-2}\alpha_{q,k}P_{q}}\geq \tau ,\;\frac{\alpha_{k-1,k:n}P_{k-1}}{1+\sum_{q=0}^{k-2}\alpha_{q,k:n}P_{q}}\geq \tau ,\;k=1,\cdots ,K,\;n=1,\cdots ,N_{k},\\ & P_{0}+P_{1}+\cdots +P_{K-1}\leq \bar{P},\\ & P_{k}\geq 0,\;k=0,\cdots ,K-1,\;\;\tau \geq 0, \end{array} \end{array}$$

which is a quadratically constrained linear programming problem. The other minimizes the power consumption with a QoS constraint, indicating that the broadcast rate should exceed the given threshold $\rho_{th}$ as follows:

(7) $$\begin{array}{c} \begin{array}{cc} \min_{P_{0},P_{1},\cdots ,P_{K-1}} & P_{0}+P_{1}+\cdots +P_{K-1}\\ s.t. & \frac{\alpha_{k-1,k}P_{k-1}}{1+\sum_{q=0}^{k-2}\alpha_{q,k}P_{q}}\geq \Gamma_{th},\;\frac{\alpha_{k-1,k:n}P_{k-1}}{1+\sum_{q=0}^{k-2}\alpha_{q,k:n}P_{q}}\geq \Gamma_{th},\;k=1,\cdots ,K,\;n=1,\cdots ,N_{k},\\ & P_{k}\geq 0,\;k=0,\cdots ,K-1, \end{array} \end{array}$$

where $\Gamma_{th}=2^{\rho_{th}}-1$. The broadcast information rate threshold $\rho_{th}$ indicates the rate at which all vehicles in the platoon are guaranteed to decode the information sent by the lead vehicle without errors. $\rho_{th}$ can be determined according to how much information should be sent by the lead vehicle and how fast the information should be transferred. Note that Equation (7) is a linear programming problem.

In the next section, we show that closed-form solutions for optimal power allocation can be derived in four-vehicle platoon communications with Λ=4, as well as suggest a relay vehicle selection approach. Deriving closed-form solutions for optimal power allocation with Λ>4 would be an interesting future research topic.

## 3. Power Allocation for 4-Vehicle Platoon Communication

In a platoon with four vehicles, the first and the last vehicles become the lead and tail vehicles, respectively. Two vehicles located in the middle of the platoon can be relay vehicles or member vehicles. Let the case where these two vehicles are selected as relay vehicles be *Case 1* with the notation of *L-R-R-T*. When only one of the two vehicles is a relay vehicle, we have *Case 2* with *L-R-M-T* and *Case 3* with *L-M-R-T*. In *Case 4* with *L-M-M-T*, the two vehicles are member vehicles.

The lead vehicle can gather the channel state information between the vehicles and perform the following power allocation and relay vehicle selection process. After the relay vehicles are chosen, the clusters are formed as described in Section 2.1, and the lead vehicle informs the power allocation result to the relay vehicles.

### 3.1. Maximize Broadcast Rate with Power Constraint

#### 3.1.1. Case 1 (L-R-R-T)

In this case, Equation (6) can be written as

(8) $$\begin{array}{c} \begin{array}{cc} \max_{P_{0},P_{1},P_{2},\tau } & \tau \\ s.t. & C1:\alpha_{0,1}P_{0}\geq \tau ,\\ & C2:\frac{\alpha_{1,2}P_{1}}{1+\alpha_{0,2}P_{0}}\geq \tau ,\\ & C3:\frac{\alpha_{2,3}P_{2}}{1+\alpha_{0,3}P_{0}+\alpha_{1,3}P_{1}}\geq \tau ,\\ . & C4:P_{0}+P_{1}+P_{2}\leq \bar{P},\\ & C5:P_{0}\geq 0,\;P_{1}\geq 0,\;P_{2}\geq 0,\;\tau \geq 0. \end{array} \end{array}$$

There is no member vehicle in this case. It is obvious that (C1) in Equation (8) yields

(9) $$P_{0}\geq \frac{\tau }{\alpha_{0,1}}.$$

Considering (C2) in Equations (8) and (9), we have

(10) $$P_{1}\geq \frac{\tau }{\alpha_{1,2}}\left(1+\frac{\alpha_{0,2}}{\alpha_{0,1}}\tau \right).$$

Similarly, we use (C3) in Equations (8)–(10) to obtain

(11) $$P_{2}\geq \frac{\tau }{\alpha_{2,3}}\left[1+\frac{\alpha_{0,3}}{\alpha_{0,1}}\tau +\frac{\alpha_{1,3}}{\alpha_{1,2}}\tau \left(1+\frac{\alpha_{0,2}}{\alpha_{0,1}}\tau \right)\right].$$

Substituting Equations (9)–(11) into (C4) in Equation (8), we have

(12) $$\bar{P}\geq \frac{\tau }{\alpha_{0,1}}+\frac{\tau }{\alpha_{1,2}}\left(1+\frac{\alpha_{0,2}}{\alpha_{0,1}}\tau \right)+\frac{\tau }{\alpha_{2,3}}\left[1+\frac{\alpha_{0,3}}{\alpha_{0,1}}\tau +\frac{\alpha_{1,3}}{\alpha_{1,2}}\tau \left(1+\frac{\alpha_{0,2}}{\alpha_{0,1}}\tau \right)\right].$$

For τ≥0, the right-hand side of Equation (12) increases with an increase in τ. As our objective is to maximize τ, we find τ to satisfy Equation (12) with equality, which yields

(13) $$a_{3}\tau^{3}+a_{2}\tau^{2}+a_{1}\tau -\bar{P}=0,$$

where

(14) $$\begin{array}{ccc} a_{3} & = & \frac{\alpha_{1,3}\alpha_{0,2}}{\alpha_{2,3}\alpha_{1,2}\alpha_{0,1}},\\ a_{2} & = & \frac{\alpha_{0,2}}{\alpha_{0,1}\alpha_{1,2}}+\frac{\alpha_{0,3}}{\alpha_{0,1}\alpha_{2,3}}+\frac{\alpha_{1,3}}{\alpha_{1,2}\alpha_{2,3}},\\ a_{1} & = & \frac{1}{\alpha_{0,1}}+\frac{1}{\alpha_{1,2}}+\frac{1}{\alpha_{2,3}}. \end{array}$$

It is guaranteed that Equation (13) has one real positive root, denoted as $\tau^{⋆}$. Then, we determine

(15) $$\begin{array}{ccc} P_{0}^{⋆} & = & \frac{\tau^{⋆}}{\alpha_{0,1}},\\ P_{1}^{⋆} & = & \frac{\tau^{⋆}}{\alpha_{1,2}}\left(1+\frac{\alpha_{0,2}}{\alpha_{0,1}}\tau^{⋆}\right),\\ P_{2}^{⋆} & = & \frac{\tau^{⋆}}{\alpha_{2,3}}\left[1+\frac{\alpha_{0,3}}{\alpha_{0,1}}\tau^{⋆}+\frac{\alpha_{1,3}}{\alpha_{1,2}}\tau^{⋆}\left(1+\frac{\alpha_{0,2}}{\alpha_{0,1}}\tau^{⋆}\right)\right]. \end{array}$$

#### 3.1.2. Case 2 (L-R-M-T)

In this case, the member vehicle belongrespectively. For the non-LOS (NLOS Subsequently, we have

(16) $$\begin{array}{c} \begin{array}{cc} \max_{P_{0},P_{1},\tau } & \tau \\ s.t. & C1:\alpha_{0,1}P_{0}\geq \tau ,\\ & C2:\frac{\alpha_{1,2}P_{1}}{1+\alpha_{0,2}P_{0}}\geq \tau ,\\ & C3:\frac{\alpha_{1,2:1}P_{1}}{1+\alpha_{0,2:1}P_{0}}\geq \tau ,\\ & C4:P_{0}+P_{1}\leq \bar{P},\\ & C5:P_{0}\geq 0,\;P_{1}\geq 0,\;\tau \geq 0. \end{array} \end{array}$$

(C1) in Equation (16) yields

(17) $$P_{0}\geq \frac{\tau }{\alpha_{0,1}}.$$

From Equation (17), (C2), and (C3) in Equation (16), we have

(18) $$P_{1}\geq \max\left\{\frac{\tau }{\alpha_{1,2}}\left(1+\frac{\alpha_{0,2}}{\alpha_{0,1}}\tau \right),\frac{\tau }{\alpha_{1,2:1}}\left(1+\frac{\alpha_{0,2:1}}{\alpha_{0,1}}\tau \right)\right\}.$$

Substituting Equations (17) and (18) into (C4) in Equation (16), we obtain

(19) $$\bar{P}\geq \frac{\tau }{\alpha_{0,1}}+\max\left\{\frac{\tau }{\alpha_{1,2}}\left(1+\frac{\alpha_{0,2}}{\alpha_{0,1}}\tau \right),\frac{\tau }{\alpha_{1,2:1}}\left(1+\frac{\alpha_{0,2:1}}{\alpha_{0,1}}\tau \right)\right\}.$$

As the right-hand side of Equation (19) increases with an increase in τ for τ≥0, we also observe that τ satisfies Equation (19) with equality. First, we compute the real positive root of

(20) $$\bar{P}=\frac{\tau }{\alpha_{0,1}}+\frac{\tau }{\alpha_{1,2}}\left(1+\frac{\alpha_{0,2}}{\alpha_{0,1}}\tau \right)$$

and confirm that the root $\tau^{⋆}$ satisfies

(21) $$\frac{\tau^{⋆}}{\alpha_{1,2}}\left(1+\frac{\alpha_{0,2}}{\alpha_{0,1}}\tau^{⋆}\right)\geq \frac{\tau^{⋆}}{\alpha_{1,2:1}}\left(1+\frac{\alpha_{0,2:1}}{\alpha_{0,1}}\tau^{⋆}\right).$$

If Equation (21) is confirmed, we can determine

(22) $$\begin{array}{ccc} P_{0}^{⋆} & = & \frac{\tau^{⋆}}{\alpha_{0,1}},\\ P_{1}^{⋆} & = & \frac{\tau^{⋆}}{\alpha_{1,2}}\left(1+\frac{\alpha_{0,2}}{\alpha_{0,1}}\tau^{⋆}\right). \end{array}$$

Next, we compute the real positive root of

(23) $$\bar{P}=\frac{\tau }{\alpha_{0,1}}+\frac{\tau }{\alpha_{1,2:1}}\left(1+\frac{\alpha_{0,2:1}}{\alpha_{0,1}}\tau \right).$$

When the root $\tau^{⋆}$ satisfies

(24) $$\frac{\tau^{⋆}}{\alpha_{1,2:1}}\left(1+\frac{\alpha_{0,2:1}}{\alpha_{0,1}}\tau^{⋆}\right)\geq \frac{\tau^{⋆}}{\alpha_{1,2}}\left(1+\frac{\alpha_{0,2}}{\alpha_{0,1}}\tau^{⋆}\right),$$

we can determine

(25) $$\begin{array}{ccc} P_{0}^{⋆} & = & \frac{\tau^{⋆}}{\alpha_{0,1}},\\ P_{1}^{⋆} & = & \frac{\tau^{⋆}}{\alpha_{1,2:1}}\left(1+\frac{\alpha_{0,2:1}}{\alpha_{0,1}}\tau^{⋆}\right). \end{array}$$

#### 3.1.3. Case 3 (L-M-R-T)

In this case, the member vehicle belongs to the cluster of the relay vehicle and is indexed as 1:1. The power allocation problem is given as

(26) $$\begin{array}{c} \begin{array}{cc} \max_{P_{0},P_{1},\tau } & \tau \\ s.t. & C1:\alpha_{0,1}P_{0}\geq \tau ,\\ & C2:\alpha_{0,1:1}P_{0}\geq \tau ,\\ & C3:\frac{\alpha_{1,2}P_{1}}{1+\alpha_{0,2}P_{0}}\geq \tau ,\\ & C4:P_{0}+P_{1}\leq \bar{P},\\ & C5:P_{0}\geq 0,\;P_{1}\geq 0,\;\tau \geq 0. \end{array} \end{array}$$

From (C1) and (C2) in Equation (26), we have

(27) $$P_{0}\geq \frac{\tau }{{\tilde{\alpha }}_{0,1}},$$

where ${\tilde{\alpha }}_{0,1}=\min\left\{\alpha_{0,1},\alpha_{0,1:1}\right\}$. Using Equation (27) and (C3) in Equation (26), we obtain

(28) $$P_{1}\geq \frac{\tau }{\alpha_{1,2}}\left(1+\frac{\alpha_{0,2}}{{\tilde{\alpha }}_{0,1}}\tau \right).$$

Substituting Equations (27) and (28) into (C4) in Equation (26), we follow the same approach described above to obtain

(29) $$\bar{P}=\frac{\tau }{{\tilde{\alpha }}_{0,1}}+\frac{\tau }{\alpha_{1,2}}\left(1+\frac{\alpha_{0,2}}{{\tilde{\alpha }}_{0,1}}\tau \right).$$

After computing the real positive root of Equation (29), we can determine $P_{0}^{⋆}$ and $P_{1}^{⋆}$.

#### 3.1.4. Case 4 (L-M-M-T)

In this case, only the lead vehicle consumes $\bar{P}$.

### 3.2. Minimizing Power Consumption with QoS constraint

#### 3.2.1. Case 1 (L-R-R-T)

The problem in Equation (7) for this case is given as

(30) $$\begin{array}{c} \begin{array}{cc} \min_{P_{0},P_{1},P_{2}} & P_{0}+P_{1}+P_{2}\\ s.t. & \alpha_{0,1}P_{0}\geq \Gamma_{th},\\ & \frac{\alpha_{1,2}P_{1}}{1+\alpha_{0,2}P_{0}}\geq \Gamma_{th},\\ & \frac{\alpha_{2,3}P_{2}}{1+\alpha_{0,3}P_{0}+\alpha_{1,3}P_{1}}\geq \Gamma_{th},\\ & P_{0}\geq 0,\;P_{1}\geq 0,\;P_{2}\geq 0. \end{array} \end{array}$$

Let us express Equation (30) in the matrix form, given as

(31) $$\begin{array}{c} \begin{array}{cc} \min_{p} & b^{T}p\\ s.t. & C1:A^{T}p\geq c^{T},\\ & C2:P_{0}\geq 0,\;P_{1}\geq 0,\;P_{2}\geq 0, \end{array} \end{array}$$

where $p={\left[P_{0}\;P_{1}\;P_{2}\right]}^{T}$, $b=1_{3}$, $c=\Gamma_{th}1_{3}^{T}$,

(32) $$\begin{array}{c} A=\left[\begin{array}{ccc} \alpha_{0,1} & -\Gamma_{th}\alpha_{0,2} & -\Gamma_{th}\alpha_{0,3}\\ 0 & \alpha_{1,2} & -\Gamma_{th}\alpha_{1,3}\\ 0 & 0 & \alpha_{2,3} \end{array}\right], \end{array}$$

and $1_{3}$ denotes a 3×1 vector with all entries equal to 1. As Equation (31) is a linear programming problem, we can obtain its dual problem given as

(33) $$\begin{array}{c} \begin{array}{cc} \max_{x} & cx\\ s.t. & C1:Ax\leq b,\\ & C2:x_{0}\geq 0,\;x_{1}\geq 0,\;x_{2}\geq 0, \end{array} \end{array}$$

where $x={\left[x_{0}\;x_{1}\;x_{2}\right]}^{T}$.

According to the duality of linear programming [28], the optimal solutions of Equations (31) and (33), which are denoted as $p^{⋆}$ and $x^{⋆}$, respectively, should satisfy

(34) $$\begin{array}{ccc} {\left(p^{⋆}\right)}^{T}\left(b-Ax^{⋆}\right) & = & 0, \end{array}$$

(35) $$\begin{array}{ccc} \left({\left(p^{⋆}\right)}^{T}A-c\right)x^{⋆} & = & 0. \end{array}$$

In Equation (34), each entry of $p^{⋆}$ is assumed to be greater than zero in this case, and $x^{⋆}$ should satisfy (C1) in Equation (33). Subsequently, we have to determine $x^{⋆}$ by $b=Ax^{⋆}$. Each entry of $x^{⋆}$ is greater than zero. Considering (C1) in Equation (31), we can observe that $p^{⋆}$ should satisfy ${\left(p^{⋆}\right)}^{T}A=c$ in Equation (35), which yields

(36) $$\begin{array}{ccc} P_{0}^{⋆} & = & \frac{\Gamma_{th}}{\alpha_{0,1}},\\ P_{1}^{⋆} & = & \frac{\Gamma_{th}}{\alpha_{1,2}}\left(1+\frac{\alpha_{0,2}\Gamma_{th}}{\alpha_{0,1}}\right),\\ P_{2}^{⋆} & = & \frac{\Gamma_{th}}{\alpha_{2,3}}\left[1+\frac{\alpha_{0,3}\Gamma_{th}}{\alpha_{0,1}}+\frac{\alpha_{1,3}\Gamma_{th}}{\alpha_{1,2}}\left(1+\frac{\alpha_{0,2}\Gamma_{th}}{\alpha_{0,1}}\right)\right]. \end{array}$$

The above approach can also be exploited in the case where the number of vehicles in a platoon is greater than four, and all vehicles located in the middle of the platoon are selected to be relay vehicles.

#### 3.2.2. Case 2 (L-R-M-T)

In this case, we have the following problem:

(37) $$\begin{array}{c} \begin{array}{cc} \min_{P_{0},P_{1}} & P_{0}+P_{1}\\ s.t. & C1:\alpha_{0,1}P_{0}\geq \Gamma_{th},\\ & C2:\frac{\alpha_{1,2}P_{1}}{1+\alpha_{0,2}P_{0}}\geq \Gamma_{th},\\ & C3:\frac{\alpha_{1,2:1}P_{1}}{1+\alpha_{0,2:1}P_{0}}\geq \Gamma_{th},\\ & C4:P_{0}\geq 0,\;P_{1}\geq 0. \end{array} \end{array}$$

From (C1) in Equation (37), we determine

(38) $$P_{0}^{⋆}=\frac{\Gamma_{th}}{\alpha_{0,1}}.$$

Using (C2) and (C3) in Equations (37) and (38), we have

(39) $$P_{1}^{⋆}=\max\left\{\frac{\Gamma_{th}}{\alpha_{1,2}}\left(1+\frac{\alpha_{0,2}\Gamma_{th}}{\alpha_{0,1}}\right),\frac{\Gamma_{th}}{\alpha_{1,2:1}}\left(1+\frac{\alpha_{0,2:1}\Gamma_{th}}{\alpha_{0,1}}\right)\right\}.$$

#### 3.2.3. Case 3 (L-M-R-T)

In this case, Equation (7) can be written as

(40) $$\begin{array}{c} \begin{array}{cc} \min_{P_{0},P_{1}} & P_{0}+P_{1}\\ s.t. & C1:\alpha_{0,1}P_{0}\geq \Gamma_{th},\\ & C2:\alpha_{0,1:1}P_{0}\geq \Gamma_{th},\\ & C3:\frac{\alpha_{1,2}P_{1}}{1+\alpha_{0,2}P_{0}}\geq \Gamma_{th},\\ & C4:P_{0}\geq 0,\;P_{1}\geq 0. \end{array} \end{array}$$

From (C1) and (C2) in Equation (40), we can obtain

(41) $$P_{0}^{⋆}=\frac{\Gamma_{th}}{{\tilde{\alpha }}_{0,1}}.$$

Using (C3) in Equations (40) and (41), we have

(42) $$P_{1}^{⋆}=\frac{\Gamma_{th}}{\alpha_{1,2}}\left(1+\frac{\alpha_{0,2}}{{\tilde{\alpha }}_{0,1}}\Gamma_{th}\right).$$

#### 3.2.4. Case 4 (L-M-M-T)

In this case, the index of the tail vehicle is 1, and the two member vehicles are indexed as 1:1 and 1:2, respectively. Subsequently, we determine $P_{0}^{⋆}=\max\left\{\frac{\Gamma_{th}}{\alpha_{0,1}},\frac{\Gamma_{th}}{\alpha_{0,1:1}},\frac{\Gamma_{th}}{\alpha_{0,1:2}}\right\}$.

### 3.3. Relay Vehicle Selection

In Section 3.1, the optimal power allocation to maximize the broadcast rate is obtained for each case. Subsequently, we compute the information rates at the vehicles as in Equation (3) to determine the broadcast rate as in Equation (4) for each case. A case to provide the maximum broadcast rate can be chosen. Similarly, we obtain the optimal power allocation to minimize the power consumption in each case in Section 3.2. We can also select the case to provide the minimum power consumption.

## 4. Numerical Evaluation

In this section, we present numerical results to validate the performance of the proposed scheme in four-vehicle platoon communications. It is assumed that four vehicles in a platoon are located in a line, as shown in Figure 1. In our simulation, we follow the path loss model for inter-vehicle mmWave channels in [16] given as

(43) $$PL\left(\hat{d}\right)=10A\log_{10}\hat{d}+C+15\frac{\hat{d}}{1000},$$

where $\hat{d}$ is the distance in meters, *A* is the path loss exponent, and *C* is a constant. For the LOS cases, *A* and *C* are given as 1.77 and 70, respectively. For the non-LOS (NLOS) cases, we set A=1.71 and C=78.6 when a single vehicle is located midway between the transmit and receive vehicles, whereas A=0.635 and C=115 are provided when two vehicles block the LOS path between the transmit and receive vehicles. Using Equation (43), the channel coefficient between vehicles is evaluated by $\sqrt{10^{-\frac{PL(\hat{d})}{10}}}g$, where *g* follows a complex Gaussian distribution with zero mean and unit variance. The noise power is $\sigma^{2}=10^{-6}$. For simplicity, the distances between adjacent vehicles are assumed to be the same as *d*.

The proposed power allocation scheme described in Section 3 is based on the assumption that self-interference at the FD relay vehicle is negligible because the transmit and receive antennas in a vehicle are well separated and the mmWave frequencies have directional properties and poor scattering characteristics. The following numerical results were also evaluated based on this assumption. When the antenna separation is not sufficient, the performance of our proposed scheme may be degraded owing to self-interference.

For comparison, we also evaluated the performance of HD relay vehicles. In Appendix A, we formulated the power allocation problems for HD relay vehicles and demonstrated that the optimal power allocation can be obtained via linear programming. For four-vehicle platoon communications with HD relay vehicles, we considered four cases, as mentioned in Section 3. We obtained the optimal power allocation for each case following the process described in Appendix A and chose the case to provide the best performance.

**Figure 2:**
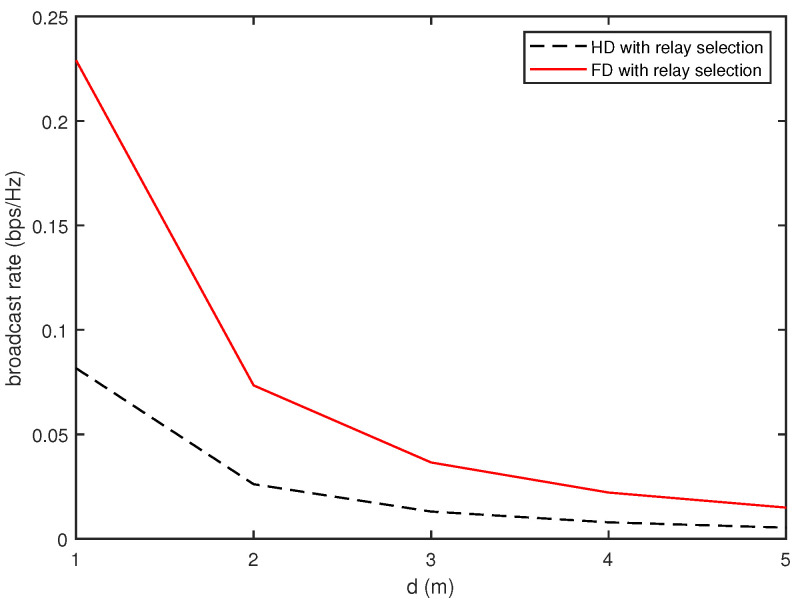
Comparison of broadcast rates as a function of *d* ($\bar{P}=10$).

**Figure 3:**
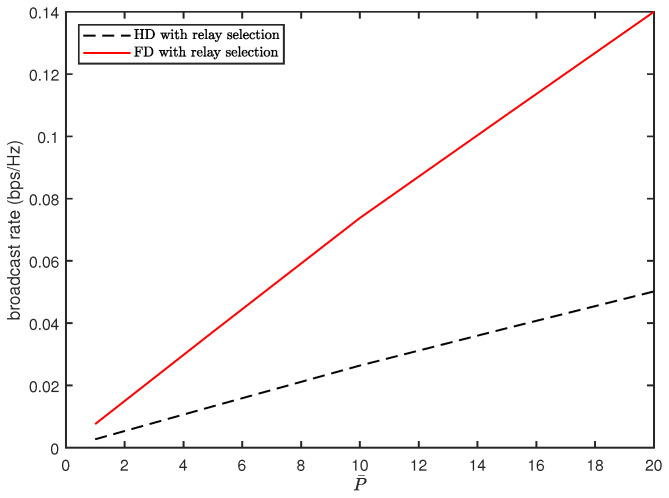
Comparison of broadcast rates as a function of $\bar{P}$ (d=2).

Figure 2 and Figure 3 present the broadcast rates achieved by the proposed FD relay vehicles with the power constraint $\bar{P}$ investigated in Section 3.1. Figure 2 compares the broadcast rate of the FD relay vehicles with that of the conventional HD vehicles for different values of *d* when $\bar{P}=10$. As the distance between the adjacent vehicles increases, the broadcast rate decreases exponentially. The FD vehicles provide better broadcast rates than the HD relay vehicles in all ranges of *d*. Figure 3 shows that the broadcast rate increases almost linearly with an increase of $\bar{P}$ for both the FD and HD relay vehicles. The FD relay vehicles also outperform the HD relay vehicles in all ranges of $\bar{P}$ and the increasing rate of the FD relay vehicles is more rapid than that of the HD relay vehicles.

**Figure 4:**
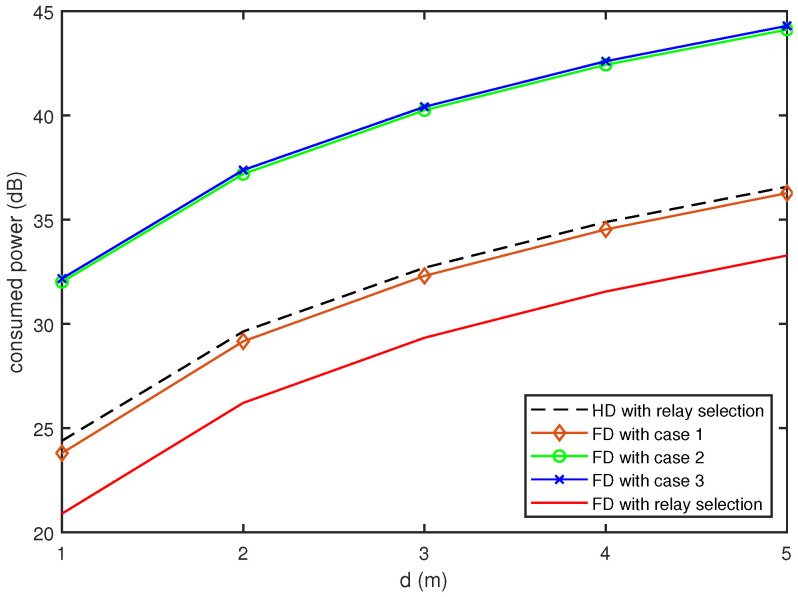
Comparison of power consumption as a function of *d* ($\rho_{th}=0.5$).

**Figure 5:**
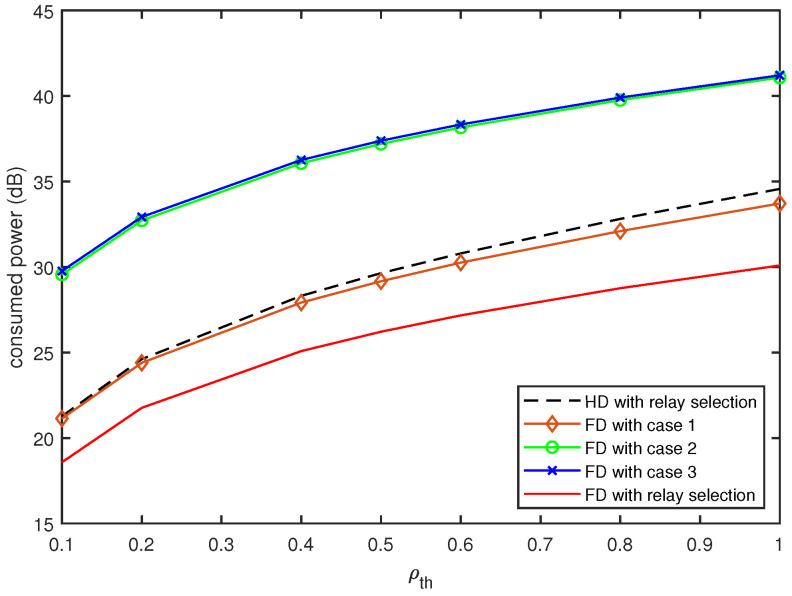
Comparison of power consumption as a function of $\rho_{th}$ (d=2).

In Figure 4 and Figure 5, we present the power consumption for the proposed FD relay vehicles with the QoS constraint $\rho_{th}$, compared with that for the conventional HD relay vehicles. To investigate the effect of the relay selection described in Section 3.3, we also evaluated the power consumption of the proposed FD relay vehicles with fixed relay selection, indicating that only one of the cases described in Section 3.2 is considered for different channel realizations. Figure 4 compares the consumed powers for the FD and HD relay vehicles for different values of *d* when $\rho_{th}=0.5$. As expected, the increase in *d* results in an increase in power consumption. When we fix the relay selection to Case 1 or Case 2, the proposed FD relay vehicles consume more power than the HD relay vehicles with the relay selection. Furthermore, the FD relay vehicle with Case 3 has marginal gain against the HD relay vehicles. However, the FD relay vehicles with the relay selection consume 3.3∼3.5 dB less power than the HD relay vehicles with the relay selection.

Figure 5 compares the power consumption as a function of $\rho_{th}$. For different values of $\rho_{th}$, the FD relay vehicles with Case 1 or Case 2 consume more power than the HD relay vehicles with the relay selection, whereas the FD relay vehicles with Case 3 consume slightly less power. As $\rho_{th}$ increases from 0.1 to 1, the power gain of the FD relay vehicle with the relay selection against the HD relay vehicles increases from 2.7 to 4.5 dB.

## 5. Conclusions

In this study, we considered mmWave vehicular platoon communications where the lead vehicle broadcasts information to all vehicles in a platoon. Considering the good isolation between transmitting and receiving antennas in a vehicle and the directional properties and poor scattering characteristics of mmWave frequencies, we suggested exploiting the FD operation at the relay vehicles. In vehicular platoon communication scenarios with relay vehicles, two power allocation problems were investigated, namely maximizing broadcast rates with a power constraint and minimizing power consumption with a QoS constraint. The closed-form solutions for optimal power allocation were derived for four-vehicle platoon communications with FD relay vehicles, and the relay vehicle selection approach was suggested. Numerical results verified that our proposed FD relay vehicles outperform conventional HD relay vehicles.

## Figures and Tables

**Table 1:** List of key notations

Symbol	Description
Λ	Number of vehicles in a platoon
*K*	Number of clusters in a platoon
$N_{k}$	Number of member vehicles in the *k*th cluster
$y_{k}$	Received signal at the *k*th relay vehicle
$y_{k:n}$	Received signal at the *k*th relay vehicle
$h_{q,k}$	Channel coefficient between the *q*th relay vehicle and the *k*th relay vehicle
$h_{q,k:n}$	Channel coefficient between the *q*th relay vehicle and the *n*th member vehicle in the *k*th cluster
$P_{0}$	Transmit power of the lead vehicle
$P_{k}$	Transmit power of the *k*th relay vehicle
$s_{0}$	Information symbol transmitted by lead vehicle
$s_{k}$	Information symbol transmitted by the *k*th vehicle
$R_{k}$	Information rate at the *k*th relay vehicle
$R_{k:n}$	Information rate at the *n*th member vehicle in the *k*th cluster
*R*	Broadcast information rate
$\bar{P}$	Overall power consumption limit
$\rho_{th}$	Broadcast information rate threshold

## Appendix A

We consider that each relay vehicle in Figure 1 operates in the HD mode. In the *k*th time slot, the *k*th relay vehicle sends information *s* to the following vehicles with k=0,1,⋯,K−1, where *K* time slots are required. In the *q*th time slot, the received signals at the *k*th relay vehicle and the *n*th member vehicle in the *k*th cluster can be given as

(A1) $$\begin{array}{ccc} y_{k}\left(q\right) & = & h_{q,k}\sqrt{P_{q}}s+z_{k}\left(q\right), \end{array}$$

(A2) $$\begin{array}{ccc} y_{k:n}\left(q\right) & = & h_{q,k:n}\sqrt{P_{q}}s+z_{k:n}\left(q\right), \end{array}$$

where q=0,1,⋯,k−1. Assuming that the vehicles perform maximal ratio combining with their received signals, we compute the information rates given as

(A3) $$\begin{array}{ccc} R_{k} & = & \frac{1}{K}\log_{2}\left(1+\sum_{q=0}^{k-1}\alpha_{q,k}P_{q}\right), \end{array}$$

(A4) $$\begin{array}{ccc} R_{k:n} & = & \frac{1}{K}\log_{2}\left(1+\sum_{q=0}^{k-1}\alpha_{q,k:n}P_{q}\right). \end{array}$$

The power allocation problem to maximize the broadcast rate with a power constraint is expressed as

(A5) $$\begin{array}{c} \begin{array}{cc} \max_{P_{0},P_{1},\cdots ,P_{K-1}} & \min\left\{\sum_{q=0}^{k-1}\alpha_{q,k}P_{q},\;\sum_{q=0}^{k-1}\alpha_{q,k:n}P_{q};\;k=1,\cdots ,K,\;n=1,\cdots ,N_{k}\right\}\\ s.t. & P_{0}+P_{1}+\cdots +P_{K-1}\leq \bar{P},\\ & P_{k}\geq 0,\;k=0,\cdots ,K-1, \end{array} \end{array}$$

which is equivalently rewritten as

(A6) $$\begin{array}{c} \begin{array}{cc} \max_{P_{0},P_{1},\cdots ,P_{K-1},\tau } & \tau \\ s.t. & \sum_{q=0}^{k-1}\alpha_{q,k}P_{q}\geq \tau ,\;\;\sum_{q=0}^{k-1}\alpha_{q,k:n}P_{q}\geq \tau ,\;k=1,\cdots ,K,\;n=1,\cdots ,N_{k},\\ & P_{0}+P_{1}+\cdots +P_{K-1}\leq \bar{P},\\ & P_{k}\geq 0,\;k=0,\cdots ,K-1,\;\;\tau \geq 0. \end{array} \end{array}$$

The power allocation problem to minimize the power consumption with a QoS constraint is also given as

(A7) $$\begin{array}{c} \begin{array}{cc} \min_{P_{0},P_{1},\cdots ,P_{K-1}} & P_{0}+P_{1}+\cdots +P_{K-1}\\ s.t. & \sum_{q=0}^{k-1}\alpha_{q,k}P_{q}\geq \Gamma_{th}^{\left(K\right)},\;\sum_{q=0}^{k-1}\alpha_{q,k:n}P_{q}\geq \Gamma_{th}^{\left(K\right)},\;k=1,\cdots ,K,\;n=1,\cdots ,N_{k},\\ & P_{k}\geq 0,\;k=0,\cdots ,K-1, \end{array} \end{array}$$

where $\Gamma_{th}^{\left(K\right)}=2^{\rho_{th}K}-1$. Both Equations (A6) and (A7) are linear programming problems. Platoon communication with three vehicles can be considered as a two-hop decode-and-forward relay network investigated in [29,30].
